# Supplementary material for: Ultraviolet Photodetecting and Plasmon-to-Electric Conversion of Controlled Inkjet-Printing Thin-Film Transistors
Source: Nanomaterials (Basel). 2020 Mar 4;10(3):458. doi: 10.3390/nano10030458 (PMC7153598; doi:10.3390/nano10030458)
Supplement: Supplementary file 1 [file nanomaterials-10-00458-s001.pdf]

Supplementary Materials

Ultraviolet Photodetecting and Plasmon-to-Electric Conversion of Controlled Inkjet-Printing Thin-Film Transistors

Cheng-Jyun Wang [1], Hsin-Chiang You [2], Jen-Hung Ou [1], Yun-Yi Chu [1] and Fu-Hsiang Ko [1,*]

[1] Department of Materials Science and Engineering, National Chiao Tung University, 1001 University Road, Hsinchu City 30010, Taiwan; cjwang.nano03g@g2.nctu.edu.tw (C.-J.W.); harveyou@hotmail.com.tw (J.-H.O.); taipei.wayne@gmail.com (Y.-Y.C.)

[2] Department of Electronic Engineering, National Chin-Yi University of Technology No. 57, Sec. 2, Zhongshan Road, Taiping District, Taichung City 41170, Taiwan; hcyou@ncut.edu.tw

* Correspondence: fhko@mail.nctu.edu.tw; Tel.: +886-3571-2121 (ext. 55803)

Received: 10 February 2020; Accepted: 26 February 2020; Published: date

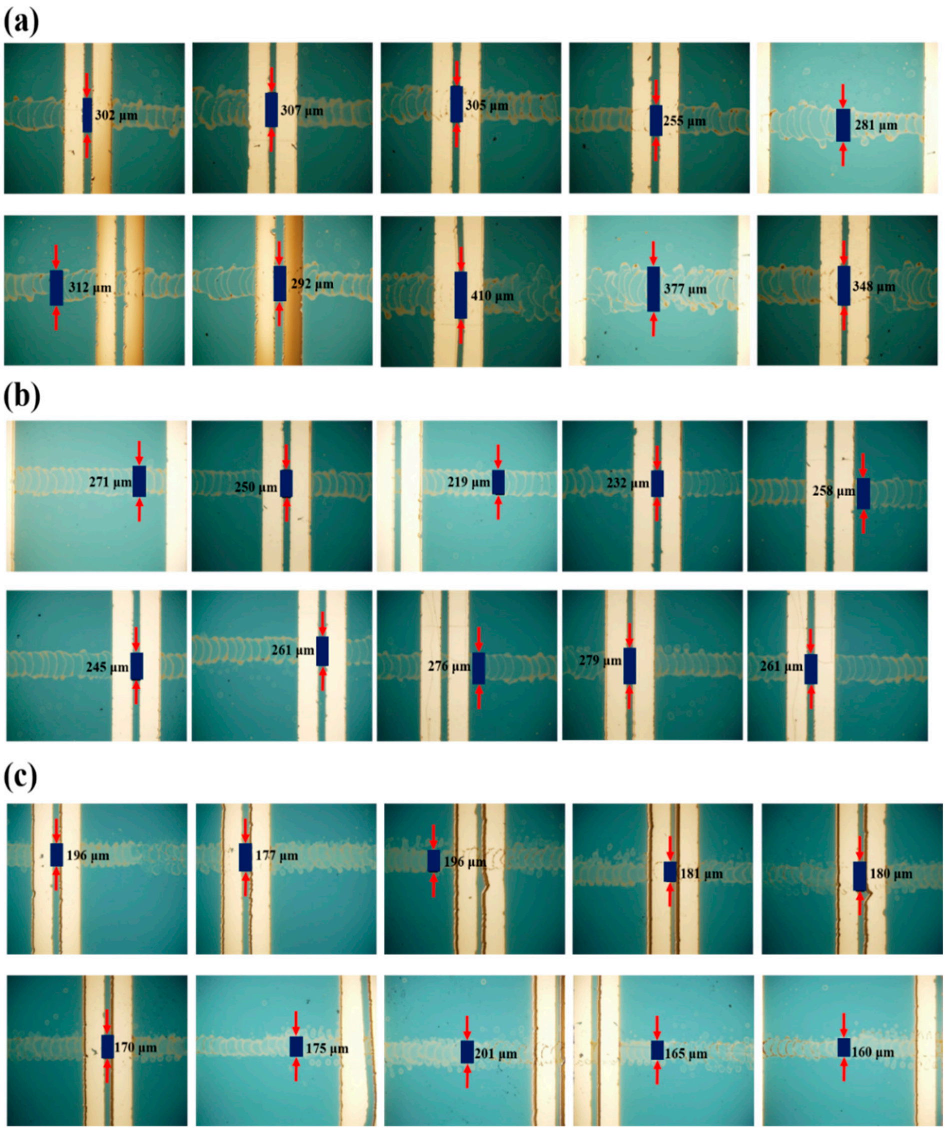

Figure S1. The optical microscope (OM) images of each single ZnO channel width by an IJP droplet with the substrate temperature at (**a**) room temperature, (**b**) 40 °C, and (**c**) 60 °C.

Table 1. The channel widths of each ZnO droplet with different temperature.

The channel widths of each ZnO droplet with different temperature (μm)										
	**Device 1**	**Device 2**	**Device 3**	**Device 4**	**Device 5**	**Device 6**	**Device 7**	**Device 8**	**Device 9**	**Device 10**
R.T.	302	307	305	255	281	312	292	410	377	348
40 °C	271	250	219	232	258	245	261	276	279	261
60 °C	196	177	196	181	180	170	175	201	165	160

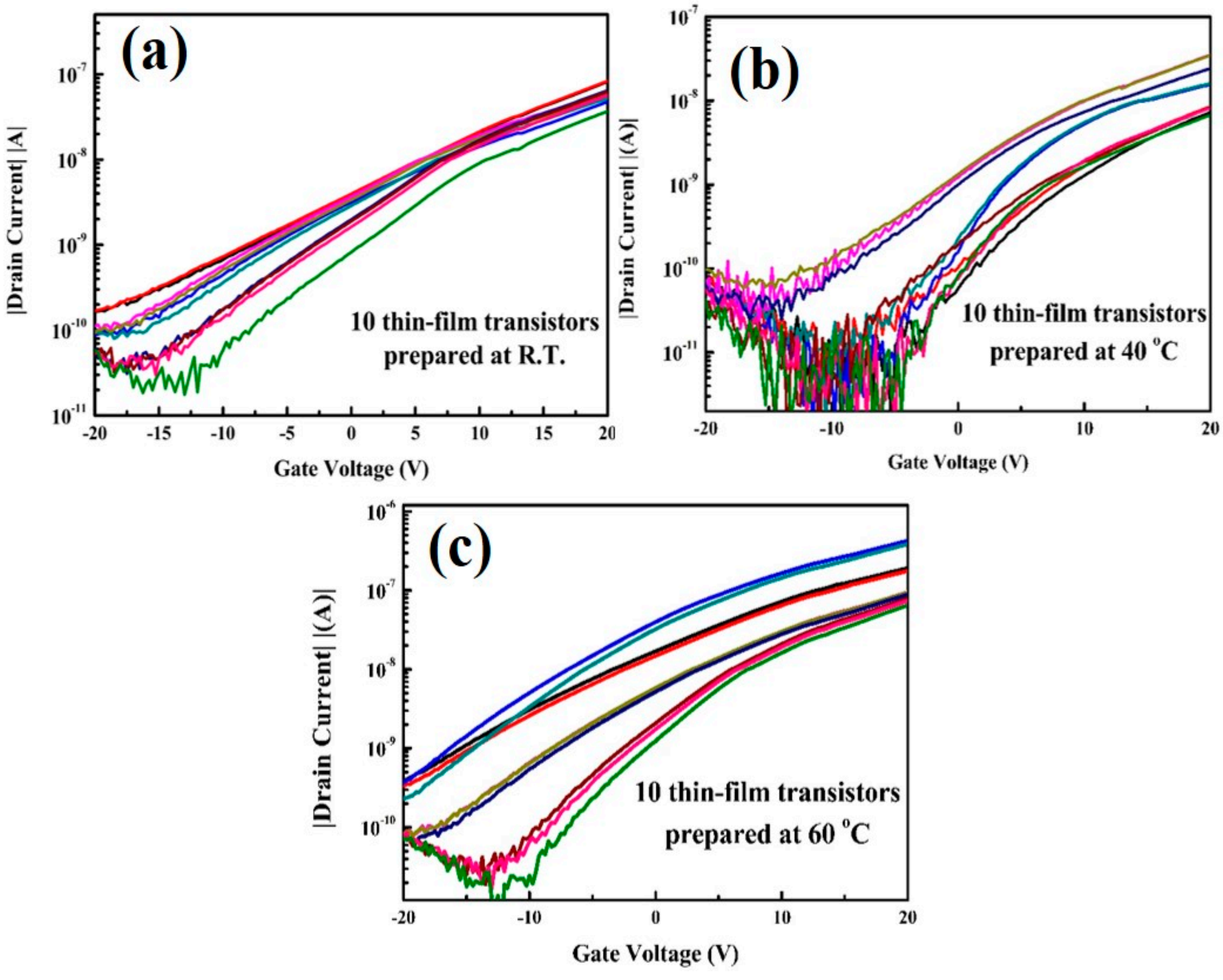

Figure S2. The typical transfer characteristics of I_{DS}-V_{GS}, where I_{DS} was measured while V_{GS} was scanned from -20 V up to 20 V at a constant V_{DS} of 5 V with the substrate temperature at (a) room temperature, (b) 40 °C, and (c) 60 °C.

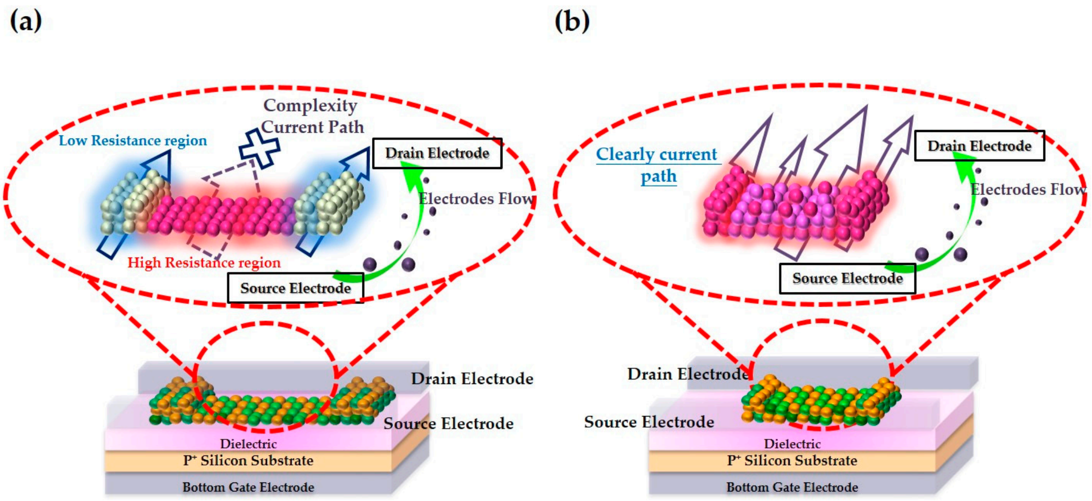

Figure S3. The ring-like channel wire widths depositing effect and the semiconductor within conduction carrier flowing behaviors. The ZnO solution inkjet droplets deposited onto the Si_3N_4 dielectric layer at (a) room temperature (R.T.), (b) at 40 ºC substrate temperature.

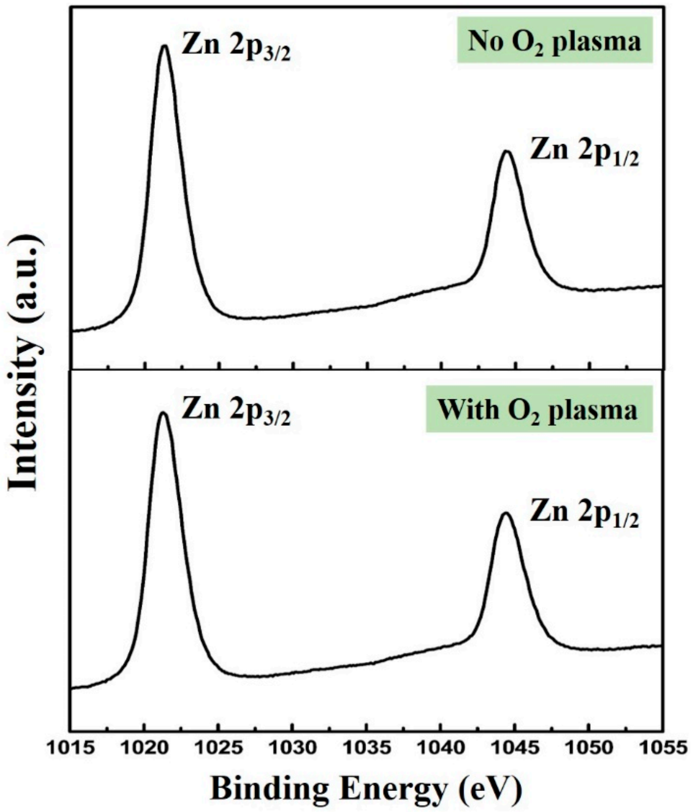

Figure S4. The XPS spectra of the Zn $2p_{3/2}$ and Zn $2p_{1/2}$ components were centered at ~1021.4 and ~1044.5 eV of ZnO films with/without oxygen plasma treatment.

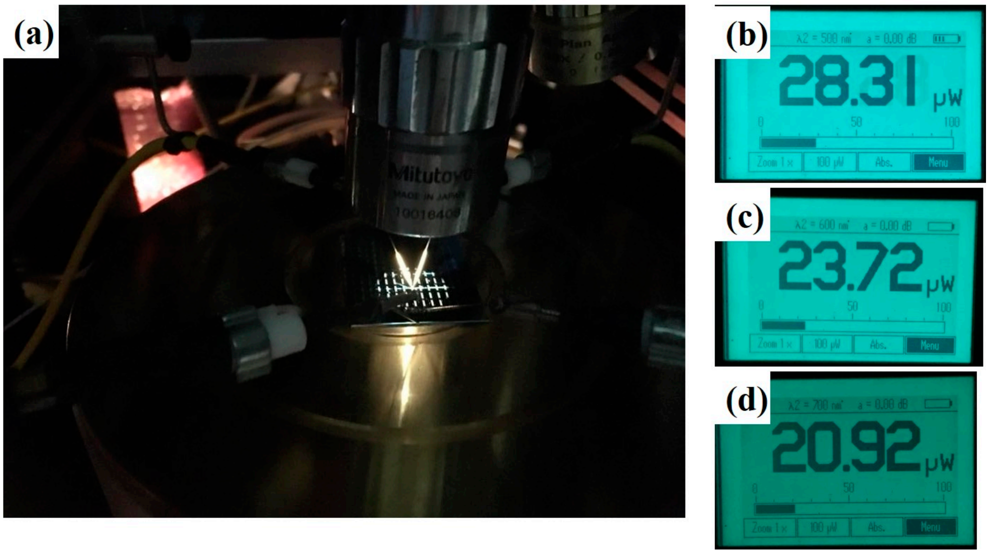

Figure S5. (a) The photograph of the ZnO-based photodetector on a semiconductor measuring the load under the Halogen lamp illumination. This Halogen lamp power at visible light wavelength of 500, 600, and 700 nm were (b) 28.31, (c)23.72, and (d) 20.92 μW.

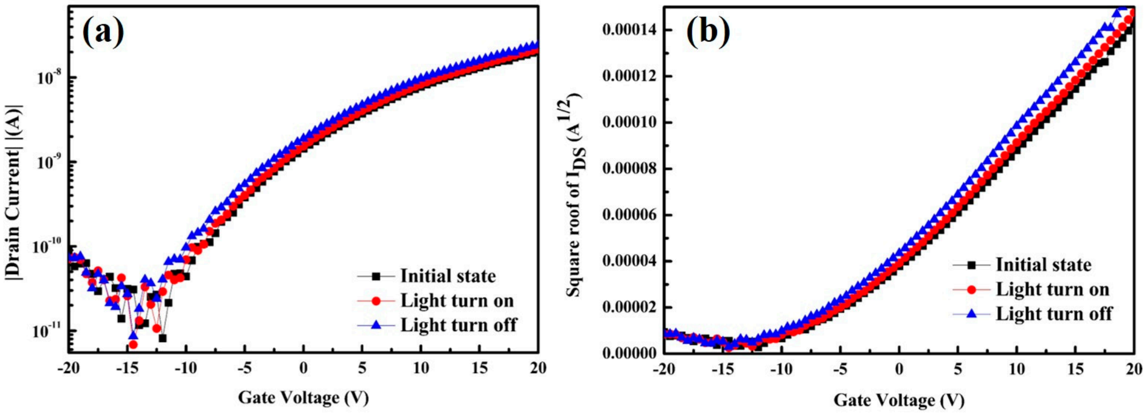

Figure S6. The electrical characteristics under the Halogen lamp illumination. (a) The I_{DS}-V_{GS} curves of the light under darkness (initial state), under light illumination and with the light turned off. (b) Square root of the electrical characteristics.

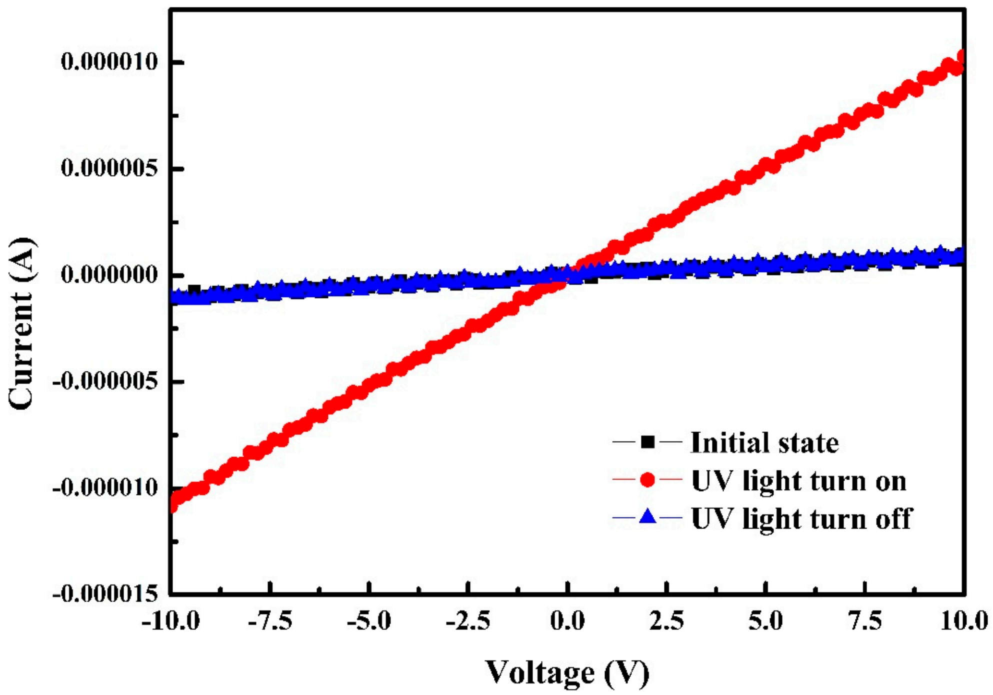

Figure S7. Current versus voltage (*I-V*) curves of the ZnO-based optoelectronic under light illumination and with the light turned off.

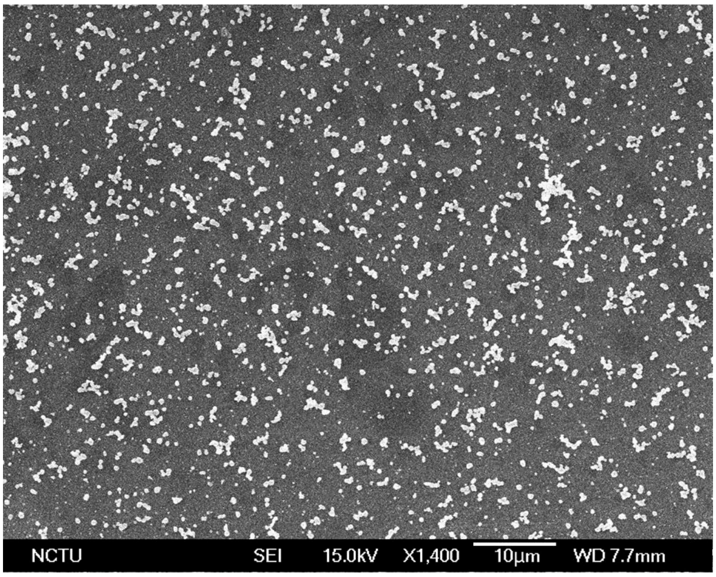

Figure S8. The SEM image of the transparent ZnO channel layer region, where AuNPs are linked at the interface between the ZnO and dielectric layer by 3-mercaptopropyltrimeth-oxysilane (MPTMS).

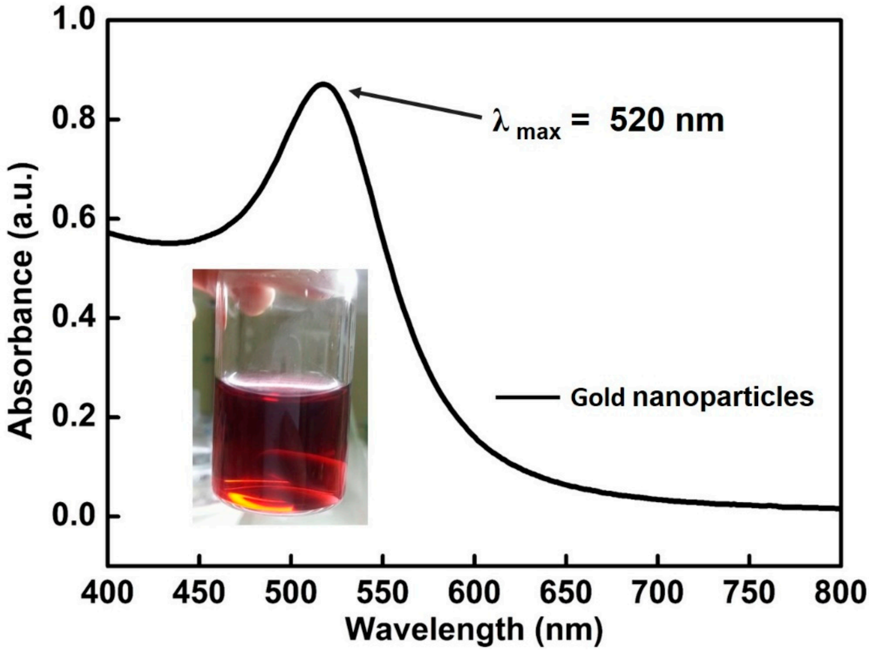

Figure S9. The UV-vis absorption spectrum of AuNPs solution. The insert picture is the photograph of AuNPs solution. The synthesis of AuNPs which we used in this study was synthesized by hydrothermal process. Then AuNPs solution was measured the absorbance with UV-vis spectroscopy. Figure shows the result solution of AuNPs which was characterized by an absorption maximum at 520 nm.
